# Impact of COVID-19 Pandemic on Sleep Quality, Stress Level and Health-Related Quality of Life—A Large Prospective Cohort Study on Adult Danes

**DOI:** 10.3390/ijerph18147610

**Published:** 2021-07-17

**Authors:** Maria Didriksen, Thomas Werge, Janna Nissen, Michael Schwinn, Erik Sørensen, Kaspar R. Nielsen, Mie T. Bruun, Karina Banasik, Thomas F. Hansen, Christian Erikstrup, Sisse R. Ostrowski, Poul J. Jennum, Henrik Hjalgrim, Henrik Ullum, Ole B. Pedersen

**Affiliations:** 1 Department of Clinical Immunology, Copenhagen University Hospital, Rigshospitalet, 2100 Copenhagen, Denmark; ioanna.nissen@regionh.dk (J.N.); michael.schwinn@regionh.dk (M.S.); erik.sorensen@regionh.dk (E.S.); sisse.rye.ostrowski@regionh.dk (S.R.O.); heul@ssi.dk (H.U.); 2 Institute of Biological Psychiatry, Mental Health Centre Sct. Hans, Mental Health Services, 4000 Roskilde, Denmark; thomas.werge@regionh.dk; 3 Department of Clinical Medicine, University of Copenhagen, 2100 Copenhagen, Denmark; 4 The Lundbeck Foundation Initiative for Integrative Psychiatric Research, iPSYCH, 2100 Copenhagen, Denmark; 5 Department of Clinical Immunology, Aalborg University Hospital, 9100 Aalborg, Denmark; k.nielsen@rn.dk; 6 Department of Clinical Immunology, Odense University Hospital, 5000 Odense, Denmark; mie.topholm.bruun@rsyd.dk; 7 Novo Nordisk Foundation Center for Protein Research, University of Copenhagen, 2100 Copenhagen, Denmark; karina.banasik@cpr.ku.dk; 8 Danish Headache Center, Glostrup Research Institute, Department of Neurology, Copenhagen University Hospital Rigshospitalet-Glostrup, 2600 Glostrup, Denmark; thomas.hansen@regionh.dk; 9 Department of Clinical Immunology, Aarhus University Hospital, 8200 Aarhus, Denmark; christian.erikstrup@skejby.rm.dk; 10 Department of Clinical Neurophysiology, Danish Center for Sleep Medicine, Copenhagen University Hospital, Rigshospitalet, 2600 Glostrup, Denmark; poul.joergen.jennum@regionh.dk; 11 Faculty of Health, University of Copenhagen, 2100 Copenhagen, Denmark; 12 Department of Epidemiology Research, Statens Serum Institut, 2300 Copenhagen, Denmark; hhj@ssi.dk; 13 Department of Hematology, Copenhagen University Hospital, Rigshospitalet, 2100 Copenhagen, Denmark; 14 Department of Clinical Immunology, Zealand University Hospital, 4600 Køge, Denmark; olbp@regionsjaelland.dk

**Keywords:** COVID-19, pandemic, epidemiology, public health, mental health, sleep

## Abstract

The everyday lives of Danish inhabitants have been affected by the COVID-19 pandemic, e.g., by social distancing, which was employed by the government in March 2020 to prevent the spread of SARS-CoV-2. Moreover, the pandemic has entailed economic consequences for many people. This study aims to assess changes in physical and mental health-related quality of life (MCS, PCS), in stress levels, and quality of sleep during the COVID-19 pandemic and to identify factors that impact such changes, using a prospective national cohort study including 26,453 participants from the Danish Blood Donor Study who answered a health questionnaire before the pandemic and during the pandemic. Descriptive statistics, multivariable linear and multinomial logistic regression analyses were applied. A worsening of MCS and quality of sleep was found, and an overall decrease in stress levels was observed. PCS was decreased in men and slightly increased in women. The extent of health changes was mainly affected by changes in job situation, type of job, previous use of anti-depressive medication and the participants’ level of personal stamina. Thus, living under the unusual circumstances that persisted during the COVID-19 pandemic has had a negative impact on the health of the general population. This may, in time, constitute a public health problem.

## 1. Introduction

As of 16 February 2021, over 35 million people have been infected with severe acute respiratory syndrome coronavirus 2 (SARS-CoV-2) in the European Union, and 812,410 have died while infected with the virus [1]. If Coronavirus disease 2019 (COVID-19) spreads uncontrollably, health care systems will be compromised.

Recent studies have reported that the prevalence of stress, anxiety, depressive disorder, and insomnia has increased during the COVID-19 pandemic [2,3].

These reports are similar to findings from other previous pandemics [4,5]. Consequently, it has been suggested that there is a risk for a psychiatric pandemic emerging post-COVID-19 [6]. Knowledge about specific health changes in different population settings during the COVID-19 pandemic is therefore imperative from a public health perspective.

The everyday lives of Danish inhabitants have been affected by the COVID-19 pandemic, e.g., by social distancing and isolation, which has proven to be an efficient measure for preventing infection [7]. On 11 March 2020, the Danish government locked down the country, urging everyone to stay home as much as possible and prohibited large social gatherings by law. Public employees not handling critical societal functions were mandated to work from home when possible. Places with a high level of social interaction, such as restaurants etc., were closed for several months (Table S1: Political interventions aimed at preventing COVID-19 spread in Denmark). These preventive measures resulted in changes in job functions, loss of income, unemployment, or worrying about such consequences. Dealing with this, while potentially worrying about one’s own health or the health of loved ones in a context of social distancing, is an unusual situation that may impact one’s general health. 

Evidence shows that social isolation affects mental health in a negative way [8] and is associated with increased mortality [9]. However, there is a lack of knowledge about the effects on physical health outcomes [8]. The feeling of loneliness increases mortality and morbidity independently of objective social isolation [10]. This could mean that the social isolation experienced during the COVID-19 pandemic does not pose a similar health risk, as it may not be accompanied by a feeling of loneliness. 

The objective of the present study was to identify health changes that occurred during the COVID-19 pandemic after the societal lock-down as well as related characteristics. This was carried out in a nationwide cohort of Danish adults using a prospective observational study design comparing health-related quality of life (HRQL), stress, and sleep quality assessed before 11 March 2020 with those assessed after this date. Subsequently, the study investigated factors impacting changes in health, including changes in work life, lifestyle and worrying about the COVID-19 pandemic, as well as the impact of personality and behavioural traits such as stamina and COVID-19 precautionary behaviours.

## 2. Materials and Methods

This study was based on data from the Danish Blood Donor study (DBDS) [11] and from Danish registers.

**Figure 1:**
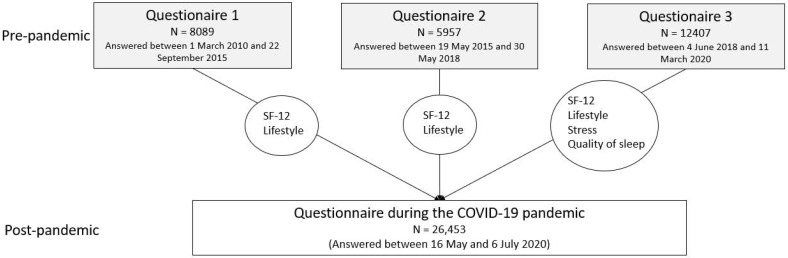
Overview of included questionnaires.

DBDS is an ongoing nationwide epidemiological cohort study [12] involving blood donors. In the years preceding the COVID-19 pandemic, a range of health-related information was collected using questionnaires. After 11 March 2020, an additional questionnaire was sent to a random sample of 75,934 participants through the governmental, personal, password protected email-system e-boks. In total, 35% of that number completed the questionnaire. In addition to the questions included in previous questionnaires, participants were asked questions directly related to their experience of the COVID-19 pandemic (Figure 1).

### 2.1. Health-Related Quality of Life

HRQL was measured using the 12-item short form health survey (SF-12), which has been validated in the general Danish population [13]. A mental component score (MCS) and a physical component score (PCS) were calculated as recommended by Quality Metric Inc. Johnston, United States [14]. In the general Danish population, the normalized mean score of PCS is 51.0 (standard deviation (SD), 8.1) and 52.8 (SD, 8.3) for MCS [13]. A higher score indicates better HRQL.

### 2.2. Level of Stress

Level of stress experienced by the participants was measured using the Cohen’s Perceived Stress Scale 10-item version (PSS-10), which was validated in the general Danish population [15]. The items were answered on a 5-point Likert scale, asking the respondents to indicate how often they experience a specific stress symptom ranging from “0 = never” to “4 = very often”, resulting in a score ranging from 0 to 40.

### 2.3. Quality of Sleep

Insomnia is characterized as a subjective experience of difficulty falling asleep and maintaining sleep or waking up too early and not being able to fall asleep again [16]. Insomnia was measured by asking respondents to report how often they experience the following symptoms: “I have difficulty falling asleep within 30 min at night”, “I wake up too early and find it difficult to fall back asleep”, and “I often wake up during the night”. Participants who experienced one or more symptoms at least three times a week were considered insomnia cases. Daytime fatigue was measured using the following three questions asking participants to indicate how often they experience the following: “I feel extremely tired during the day”, “I have an irresistible urge to sleep when at work/school”, “I have an irresistible urge to sleep in my spare time”. Again, participants who experienced one or more symptoms at least three times a week were registered as cases. Finally, participants were asked to report whether they experience restlessness in their legs during sleep. For this symptom, cases were classified as those who reported restless legs at least three times a week.

### 2.4. Characteristics Potentially Affecting the Extent of Health Impact

Information on smoking and body mass index (BMI) (weight in kg/height in cm^2^) was collected both before and during the COVID-19 pandemic. Moreover, participants reported how many people they were living with during the pandemic, including the ages of these. Participants were also asked about changes in their work life during the pandemic. The personality trait stamina was assessed using nine questions concerning self-perception. The applied items were based on the Connor–Davidson Resilience (CD) scale, which has been validated in several population settings [17]. We assessed the personality trait stamina using part of the CD-scale. Each item on the CD-scale was answered on a 5-point Likert scale with a summarized score ranging from 0 to 45. The higher the score, the higher the personal level of stamina (Table S2: Stamina scale).

Using pseudonymized versions of the unique civil Danish registration numbers [14], individual level data were collected from the Danish National Registers. We collected information on latest graduated educational level and previous filled prescriptions for anti-depressive drugs (Anatomical Therapeutic Chemical classification codes: N06A). Finally, information on dates for blood donation was obtained from the National Blood Bank Register.

### 2.5. Statistics

Changes in stress level, insomnia, daytime fatigue, health-related quality of life, BMI, and smoking status were investigated using descriptive statistics. *t*-tests were applied for normally distributed data (stress score) and Wilcoxon Signed Rank Tests were applied for non-normally distributed data (health-related quality of life, BMI). Dichotomous traits were investigated using chi-square tests (smoking status, insomnia, daytime fatigue) (for crude risk estimate see Table S3: Linear regression analyses). Identification of characteristics associated with decrease or increase in mental or physical health characteristics, and Table S4: Multinomial logistic regression analyses displaying relative risk ratios (RRR). Identification of characteristics associated with developing a poor quality of sleep phenotype or becoming free of a poor quality of sleep phenotype).

Subsequently, characteristics affecting changes in health were investigated. For continuous outcomes, this was carried out using multivariable linear regression analyses where delta-values for changes in MCS, PCS, and stress score were included as outcome variables in separate models. A delta-value of 0 indicated no change between scores before and during the pandemic, and negative and positive delta-values indicated decreasing and increasing scores, respectively. Delta-values were approximately normally distributed and were therefore not transformed before analyses. For sleep traits (insomnia, daytime fatigue, and restless legs during sleep) impacting characteristics were identified using multinomial logistic regression models where the outcome variables were computed as categorical variables with 0/reference = “no change”, 1 = “from case before the pandemic to non-case during”, and 2 = “from non-case before pandemic to case during” indicating the individual change in sleep. Analyses were conducted separately for men and women because of a priori knowledge of sex-related differences in the investigated outcomes [18,19,20,21,22]. Cross-sectional multivariable linear and logistic regression analyses identifying factors impacting current MCS, PCS, stress score, insomnia, daytime fatigue and restless legs during sleep are displayed in Table S5: Linear regression analyses. Identification of characteristics associated with physical and mental health characteristics measured during the COVID-19 pandemic, and Table S6: Logistic regression analyses. Identification of characteristics associated with quality of sleep measured during the COVID-19 pandemic.

### 2.6. Follow-Up Analyses

To account for the healthy donor effect and potential seasonal effects on self-perceived health [18,19,20,21,22,23,24], regression analyses were subsequently adjusted for number of days since last blood donation and for season of the year that they answered the first questionnaire. 

## 3. Results

Both positive and negative changes in health characteristics occurred among the participants since the onset of the COVID-19 pandemic. The presented risk estimates were derived from the complete statistical models including all potential covariates plus individual time intervals between answering the questionnaire before the pandemic and the one during (Table 1).

### 3.1. Distribution of Characteristics Potentially Affecting a Change in Health during the COVID-19 Pandemic

In total, 12,150 men and 14,303 women answered questionnaires both before and during the pandemic (Figure 1). Distribution of characteristics potentially affecting changes in health during the pandemic are displayed in Table 1.

### 3.2. Comparing Health Characteristics before the COVID Pandemic with Those During

Compared with baseline estimates, MCS was lower for both sexes during the COVID-19 pandemic, while PCS was lower in men and slightly higher in women. The mean individual change in MCS was −1.29 (SD, 7.08) for men and −1.44 (SD, 8.35) for women. For PCS, this was −0.29 (SD, 5.35) for men and −0.01 (SD, 5.91) for women. For stress score it was −1.21 (SD, 4.95) for men and −1.27 (SD, 5.70) for women. The proportion of smokers decreased. On average, there was an increase in BMI and in the proportion of participants suffering from insomnia, daytime fatigue and restless legs during sleep (Table 2).

### 3.3. Changes in Mental and Physical Health

#### 3.3.1. Factors Associated with Experiencing a Change in Mental Health-Related Quality of Life

The most prominent factor associated with a decreased MCS was losing one’s job during the pandemic for both sexes. Other factors associated with a decreased MCS among men were wearing disposable masks around people, working or studying from home, having experienced a change in work situation, living with another adult and avoiding travels outside one’s own region or country. The only other factor associated with decreased MCS in women was smoking.

For both sexes, having a previously filled prescription for anti-depressive medication was associated with increased MCS. For each additional point on the stamina score, both men and women experienced an average MCS increase of 0.15 points. Men who reported that they were living with one or more children or feeling worried about the pandemic were likely to have experienced a slight increase in MCS. The same was observed for increased BMI among women (Table 3).

#### 3.3.2. Factors Associated with Experiencing a Change in Physical Health-Related Quality of Life

Among men, the most prominent factor associated with reduced PCS was working in the sales or service industry. The same was observed among women employed as unskilled workers. Among men, no factors were independently associated with increased PCS, while losing one’s job during the pandemic was associated with an increase in women. For both sexes, an increase in BMI was associated with a decreased PCS. The same was observed for those reporting that they were staying home as much as possible during the pandemic. Using disposable handkerchiefs was associated with decreased PCS for men, while the same was true for wearing masks among women. Finally, women who reported that they had limited their use of public transportation had an increased probability of improved PCS (Table 3).

#### 3.3.3. Factors Associated with Experiencing a Change in Stress Level

Higher stamina score was the strongest independent predictor for decreased stress level among women. A higher stamina score was also associated with decreased stress in men; however, working in the sales or service industry was the strongest predictor for decreased stress in this group. Both men and women working in administration or other office jobs when the pandemic began were more likely to have experienced decreased stress level compared to those working in academia (Table 3).

### 3.4. Change in Quality of Sleep

#### 3.4.1. Factors Associated with Either Developing or Becoming Free from Insomnia

Among men, working as an unskilled worker was associated with the highest RRR for developing insomnia (RRR = 2.10, *p* = 0.004), while having filled a prescription for anti-depressive medication previously was the strongest predictor in women (RRR = 1.37, *p* = 0.032). An increase in stamina appeared to be protective against developing insomnia in both sexes, and it was associated with an increased probability of becoming free from insomnia in women suffering from insomnia before the pandemic. Living with school-aged children seemed to decrease the probability of becoming free from insomnia among both sexes. Having a long-length education (≥5 years post high school) was associated with an increased probability of becoming free from insomnia in men. Finally, undertaking the COVID-19 precautionary measure of coughing or sneezing in one’s elbow was associated with a decreased risk of developing insomnia in women (Table 4).

#### 3.4.2. Factors Associated with Either Developing or Becoming Free from Daytime Fatigue

Men who reported losing their jobs during the pandemic had a more than three times increased risk of developing daytime fatigue (RRR = 3.51, *p* = 0.008). An increased risk was also observed for men who were employed as skilled or unskilled workers, and who reported that they use disposable handkerchiefs to avoid becoming infected with SARS-CoV-2. Among women, smoking and limiting use of public transportation increased the risk of developing daytime fatigue. 

Men suffering from daytime fatigue before the pandemic were more likely to become free of it if they were working a part-time job compared to working full time. Increased BMI and avoiding places where many people gather also increased the chance of becoming free from daytime fatigue in men. For women who were suffering from daytime fatigue before the pandemic, staying home as much as possible increased the probability of becoming free from it. Finally, an increase in length of education was associated with decreased risk of experiencing a change in daytime fatigue-status among both sexes (Table 4).

#### 3.4.3. Factors Impacting Change in Experience of Restless Legs during Sleep

Having an education of at least five years post high school was the most protective risk factor against developing restless legs during sleep among women. After this was working in administration or another office job. A higher stamina score was associated with a decreased risk of experiencing a change in restless legs case-status in both sexes, while higher BMI increased the risk of change. Thus, either developing or becoming free from restless legs during sleep was dependent on case-status when the pandemic began. In women, being employed as an unskilled worker was associated with a more than four times increased risk of becoming free from restless legs compared to those suffering from it when the pandemic began. Similarly, an increased risk of becoming free from it was observed for women who reported that they had been feeling worried about the pandemic and socially cut-off (Table 4). Follow-up analysis showed that those who experienced an increased PCS were less likely to develop restless legs if they were non-cases before the pandemic, while those who were cases before were more likely to become free from it if their PCS increased.

The observed risk estimates did not change when information on time since last blood donation and season of the year for answering the first questionnaire was included as covariates in the statistical models.

## 4. Discussion

This study brings important new knowledge about potential health consequences derived from the COVID-19 pandemic. Here we report evidence of overall worsening of MCS and quality of sleep and an overall improvement in stress levels. Moreover, a decreased PCS was observed in men, while an increase was observed in women. These findings are in line with those made in other studies that were conducted during the COVID-19 pandemic and during previous pandemics [2,3,6].

As of 11 March 2020, the Danish government implemented a national societal lock-down. Public employees were sent home, some with the possibility of working from home. Social activities in public places were prohibited by law, while social gatherings in private settings were strictly advised against. The general stress burden in Denmark has been increasing in recent years [25]. Part of this may be attributed to a combination of personal and societal expectations to oneself, possibly in terms of workload. The preventive measures taken to slow down the spread of COVID-19 therefore provided a unique opportunity to study what happens when part of the population is relieved of this burden, at least to some extent. The fact that we observed a generally decreased stress level during the pandemic indicates that the prevalence of stress disorders might be reduced by changes in the societal structure aimed at reducing the experienced workload. Moreover, we found that men working in the sales or service industry were most likely to have experienced reduced stress level, which may be caused by most retail stores being closed during the lock-down and that the stores being open (grocery stores) had a reduced number customers during this period. In line with this, it was observed that those who had previously been treated with anti-depressive medication were more likely to have experienced an increase in MCS. 

Further, the present study shows that men who were living with children during the pandemic were more likely to have experienced an increase in MCS. This supports previous findings. In a recently published narrative review investigating the effect of the COVID-19 pandemic on mental health, it was shown that families frequently reported that the mandatory social isolation produced a feeling of cohesion and partnership as well as a better understanding of which values that should be prioritized for their family [26].

It is noteworthy that we also found that having used anti-depressive medication previously was associated with a lower MCS compared to not having a previous prescription (factors associated with health characteristics measured during the COVID-19 pandemic (i.e., not changes in health characteristics) are displayed in Tables S5 and S6). Thus, even though individuals with previous use of anti-depressive medication were likely to have a lower MCS than the rest of the participants, they were also more likely to have experienced an increase in their MCS during the COVID-19 pandemic. This, again, may be interpreted as a result of mentally vulnerable individuals potentially benefiting from being relieved of social and/or work pressure.

Contrary to this, in a Italian population setting, it was found that previous use of psychiatric medications was associated with increased depression and anxiety during the COVID-19 pandemic [27]. This is likely to be explained by the fact that the Italian study was cross-sectional. Thus, the study showed increased prevalence of mental disorders in those with previous medication use compared to those without. The prospective design of the present study allowed us to identify the direction of change in MCS among those with previous medication use.

Social contact is important for a healthy cognitive, emotional, endocrine and immune development in humans [28]. It has even been reported that humans can feel a craving for social contact similar to that of food [29]. In addition to the reduced social contact, the societal lock-down was also accompanied by economic decline and a compromised healthcare system. The reduced MCS, PCS and sleep quality is highly concerning since these factors are predictors of subsequent morbidity and mortality [30]. 

Furthermore, good sleep quality has been associated with lower stress [31]. In the present study, a general increase in sleep problems and a decrease in stress was found. When investigating our data further, a mean individual decrease of 1.47 (SD, 5.16) points in stress score was observed for those without daytime fatigue during the pandemic, while a mean increase in stress score of 1.37 (SD, 6.75) was observed for those with. Similarly, a smaller decrease in stress score was observed among those with insomnia or restless legs during sleep compared to among those without (data not shown). This suggests that the link between quality of sleep and stress level also applies in this study population. In this regard, it might be speculated that the decreased stress level mitigated the extent of reduction in sleep quality. Similarly, it was previously found that increased stress level on the PSS-10 stress scale is associated with decreased MCS on the SF-12 scale [32]. This, again, may suggest a mitigation of the MCS reduction due to reduced stress—or vice versa. The observed decrease in the proportion of smokers may be caused by knowledge of smoking exacerbating COVID-19 disease. Increased BMI was observed. This, however, is likely to display a tendency in time as increases in BMI has been observed in recent years.

When data for this study were collected, the Danish population was not mandated to wear masks. Therefore, the fact that wearing masks appear to be associated with the second largest decrease in MCS among men could be a result of an extraordinary personal worry of SARS-CoV-2 infection. Furthermore, we observed that women who lost their jobs during the pandemic were more likely to have experienced a reduced MCS and an increased PCS. The reason for this is not clear; it may be that women react by practising physical exercise when their MCS drops. A higher stamina score was associated with decreased PCS among women, which is also contrary to what was found for MCS in women. 

With the size of the cohort and the prospectively collected health information, the DBDS database represents a unique opportunity to investigate the impact of the COVID-19 outbreak on public health. The study focuses on a healthier subgroup of the general population consisting of active or previous blood donors [18]. The study could therefore be impacted by healthy blood donor bias [23]. Because we focus on factors impacting changes in HRQL, stress, and sleep quality within this cohort, the concern for bias is limited. In line with this, it is known that MCS increases and PCS decreases with age in the Danish population [32]. We do not suspect that age has affected the findings, as analyses were adjusted for age and number of days between answering the questionnaire before the pandemic and the one during. Additionally, when adjusted for time since last blood donation and season of the year for answering the questionnaire, the results did not change. Because the donors are generally healthy, it is plausible that the observed health changes may not only be a result of fear for SARS-CoV-2 infection, but also a result of the societal restrictions and the consequences for social interaction and employment. However, it is impossible to decipher to what extent the observed health changes were due to the pandemic rather than just a fluctuation over time affected by numerous factors. 

The use of scales that have been validated in the Danish language [13,15] is a methodological strength of the study. The lack of objective measures for assessing sleep quality is a limitation to this study. Furthermore, the applied questions were not validated in the Danish language. However, since diagnosing insomnia in clinical practice is primarily based on subjective reports and since the applied questions were based on a validated scale [33] and chosen in corporation with a sleep expert, we consider them to be adequate for this study. Finally, it should be noted that Denmark has a public healthcare system ensuring equal and free access for all citizens and that the Danish government provided monetary support for unemployed citizens and businesses who struggled during the lock-down. It is likely that this impacts the magnitude of health changes and should therefore be considered when comparing health changes across countries.

## 5. Conclusions

To conclude, to our knowledge this is the first study to investigate changes in MCS, PCS, stress level and quality of sleep during the COVID-19 pandemic by applying a prospective study design. The findings indicate that a subgroup of mentally vulnerable individuals, prevalent among blood donors, may have benefitted from mandated social distancing. Moreover, this study displays overall negative health changes during the COVID-19 pandemic. Because MCS, PCS and quality of sleep are all associated with later comorbidity and mortality rates, it is possible that the COVID-19 pandemic, in addition to its devastating direct influence on society, will also cause reduced general public health over time. However, the effect and timeline for such a potential crisis remain unclear.

## Figures and Tables

**Table 1:** Distribution of characteristics potentially impacting change in health during the COVID-19 pandemic.

		Men (*n* = 12,150)	Women (*n* = 14,303)	N Missing
Age during the COVD pandemic (median, IQR)		52.8 (41.0; 62.7)	49.2 (35.9; 59.2)	-
Living with child(ren) (% yes)				173
	Below school age (<5 years old)	1.3	1.3	
	School age (5–18 years old)	72.7	70.0	
Living with another adult (% yes)		73.3	68.3	99
Ever been prescribed anti-depressive medication (% yes)		7.6	12.2	
Educational level (%)				
	Elementary school	9.5	9.7	
	High school or vocational course	47.1	42.9	
	Short length education	9.0	6.5	
	Medium length education	18.3	28.6	
	Long length education	16.1	12.1	
Occupation (% yes)				9
	Full time job	68.1	58.9	
	Part time job	2.8	11.5	
	Self employed	4.9	2.6	
	Student	4.1	8.3	
	Stay-at-home by choice	0.2	0.7	
	Unemployed	1.9	2.3	
	Long-term sick leave/parental leave	0.5	1.9	
	Retired	16.6	12.7	
	Other	1.0	2.1	
Type of job (% yes)				6221
	Unskilled worker	4.2	3.7	
	Skilled worker	18.4	5.4	
	Sales/service	6.8	5.3	
	Administration/office work	20.4	31.6	
	Academic	40.6	43.0	
	Other	9.6	11.5	
Specific COVID-precautions (% yes)				-
	Frequently washing hands	91.2	91.3	
	Coughing or sneezing in the elbow	78.7	83.1	
	Using disposable handkerchiefs	11.8	20.9	
	Wearing disposable masks	1.3	2.2	
	Avoiding handshakes	91.6	92.3	
	Stopped greeting people with hugs and/or kisses on both cheeks	81.7	85.8	
	Limiting use of public transportation	32.8	36.7	
	Avoid places where many people gather	62.8	72.5	
	Staying home	29.5	33.8	
	Working from home	36.3	33.3	
	Avoiding travels outside one’s own country or region	57.9	64.5	
	Take none of the above measures	1.9	0.8	
Change in work situation (%)				5432
	I work/study from home	27.2	29.8	
	I was sent home without being able to work	3.2	3.2	
	I have taken a vacation or a leave of absence	0.6	0.3	
	I was let go	1.3	1.2	
	I still go in to work, but my work situation has changed	17.7	19.1	
	My work situation has not changed	50.0	46.4	
Feeling worried about the COVID pandemic and cut-off from social experiences (% yes)				96
	Never or sometimes	87.6	90.4	
	Most of the time	12.4	9.60	
Stamina score (IQR)		31 (27; 34)	29 (26; 33)	401

**Table 2:** Comparing population health characteristics before the COVID-19 pandemic with levels during the pandemic.

Characteristic		Men (*n* = 12,150)		*p*	Women (*n* = 14,303)		*p*	N Missing
		Before	During		Before	During		
Health-related quality of life (median (IQR))								504
	Mental component score	52.8 (50.2; 56.7)	52.6 (49.2; 54.8)	<0.001	52.6 (48.3; 55.5)	51.8 (46.5; 54.5)	<0.001	
	Physical component score	56.4 (54.2; 57.6)	56.4 (53.6; 57.7)	0.0015	56.4 (53.9; 57.7)	56.6 (53.6; 57.9)	<0.001	
Smoking status (% yes)		12.1	10.3	<0.001	12.6	10.9	<0.001	133
Body mass index (median (IQR))		25.6 (23.7; 28.1)	25.7 (23.8; 28.1)	<0.001	24.5 (22.3; 27.7)	24.8 (22.5; 28.3)	<0.001	209
		Men (*n* = 5790)			Women (*n* = 6617)			
Quality of sleep (%)								
	Insomnia	26.3	28.8	0.001	27.6	30.4	<0.001	
	Daytime fatigue	4.01	6.78	<0.001	5.61	10.9	<0.001	
	Restless legs during sleep	3.01	6.15	<0.001	2.92	7.62	<0.001	
Mean stress score (SD)		9.09 (5.09)	7.82 (5.59)	<0.001	10.7 (5.64)	9.28 (6.27)	<0.001	603

*p* values were calculated using Wilcoxon Signed Rank test for the continuous variables, whereas a chi-squared test was used to calculate the *p* values for difference in the binary variables.

**Table 3:** Linear regression analyses. Identification of characteristics associated with decrease or increase in mental or physical health characteristics (displaying risk estimates for covariates with a *p* value < 0.05 in the complete model).

		Men (*n* =12,150)		Women (*n* =14,303)	
		Coef. (95% CI)	*p*	Coef. (95% CI)	*p*
Outcome: Change in mental component score ^×^					
Change in work situation					
	My work situation has not changed (reference)	-	-	-	-
	I still go into work, but my work situation has changed	−0.498 (−0.906–−0.091)	0.017		
	I work/study from home	−0.765 (−1.207–−0.322)	0.001		
	I was let go	−1.702 (−2.988–−0.416)	0.009	−2.039 (−3.606–−0.471)	0.011
Stamina score		0.154 (0.125–0.184)	<0.001	0.148 (0.115–0.180)	<0.001
Ever been prescribed anti-depressive medication		0.759 (0.177–1.342)	0.011	0.781 (0.249–1.314)	0.004
Living with child(ren)					
	No (reference)	-	-	-	-
	School age (5–18 years old)	0.423 (0.067–0.791)	0.020		
Living with another adult (yes vs. no)		−0.688 (−1.044–−0.331)	<0.001		
BMI during the pandemic (continuous)				0.039 (0.004–0.076)	0.031
Feeling worried about the COVID pandemic and cut-off from social experiences (most of the time vs. never or sometime)		0.536 (0.099–0.972)	0.016		
Smoking during the pandemic (yes vs. no)				−0.702 (−1.250–−0.153)	0.012
COVID−19 precautionary behaviour (yes vs. no)					
	Wearing disposable masks	−1.535 (−2.811–−0.259)	0.018		
	Avoiding travels outside one’s own country or region	−0.385 (−0.711–−0.059)	0.021		
	Outcome: Change in physical component score ^×^				
Change in work situation					
	My work situation has not changed (reference)	-	-	-	-
	I was let go			1.437 (0.326–2.548)	0.011
Job type					
	Academic (reference)	-	-	-	-
	Sales/Service	−0.648 (−1.166–−0.131)	0.014		
	Unskilled worker			−1.058 (−1.798–−0.319)	0.005
Stamina score				−0.028 (−0.051–−0.005)	0.018
Educational level					
	Elementary school (reference)	-	-	-	-
	Long length education			−0.766 (−1.425–−0.107)	0.023
BMI during the pandemic (continuous)		−0.052 (−0.084–−0.021)	0.001	−0.047 (−0.072–−0.021)	<0.001
Specific COVID-precautions (yes vs. no)					
	Using disposable handkerchiefs	−0.427 (−0.843–−0.015)	0.042		
	Wearing disposable masks			−0.825 (−1.596–−0.055)	0.036
	Limiting use of public transportation			0.283 (0.006–0.561)	0.045
	Staying home	−0.292 (−0.575–−0.008)	0.044	−0.341 (−0.628–−0.054)	0.020
Outcome: Change in stress score ^×^					
		Men (*n* = 5790)		Women (*n* = 6617)	
Job type					
	Academic (reference)	-	-	-	-
	Administration/Office work	−0.474 (−0.889–−0.058)	0.026	−0.602 (−1.066–−0.138)	0.011
	Sales/Service	−0.795 (−1.442–−0.148)	0.016		
Stamina score (continuous)		−0.094 (−0.124–−0.065)	<0.001	−0.102 (−0.135–−0.069)	<0.001

^×^ changes in scores were calculated by subtracting the score before the COVID-19 pandemic from the score during. A negative score represented a decrease, while a positive score represented an increase. Analyses were adjusted for age, number of days between answering the questionnaire before the pandemic and the one during, change in work situation during the pandemic, occupation, type of job, stamina score, previous use of anti-depressive medication, cohabitation (living with children and living with another adult), educational level, worry about the pandemic, smoking during the pandemic, body mass index during the pandemic, and COVID-19 precautionary behaviours. OBS: for risk estimates only adjusted for age and time interval between answering the questionnaire before the COVID-19 pandemic and the one after, see Table S3.

**Table 4:** Multinomial logistic regression analyses displaying relative risk ratios (RRR). Identification of characteristics associated with developing a poor quality of sleep phenotype or becoming free of a poor quality of sleep phenotype (displaying risk estimates for covariates with a *p* value < 0.05 in the complete model).

		Men (*n* = 5790)				Women (*n* = 6617)			
		From Non-Case to Case		From Case to Non-Case		From Non-Case to Case		From Case to Non-Case	
		RRR (95% CI)	*p*	RRR (95% CI)	*p*	RRR (95% CI)	*p*	RRR (95% CI)	*p*
Outcome: Insomnia (no change = reference)									
Job type									
	Academic (reference)	-	-	-	-	-	-	-	-
	Unskilled worker	2.10 (1.26–3.49)	0.004						
Stamina score (continuous)		0.96 (0.94–0.98)	<0.001			0.97 (0.95–0.99)	0.002	1.03 (1.01–1.05)	0.009
Ever been prescribed anti-depressive medication						1.37 (1.03–1.83)	0.032		
Living with child(ren)									
	No (reference)	-	-	-	-	-	-	-	-
	School age (5–18 years old)			0.65 (0.51–0.81)	<0.001			0.69 (0.55–0.87)	0.002
Educational level									
	Elementary school (reference)	-	-	-	-	-	-	-	-
	Long length education			1.75 (1.05–2.93)	0.033				
Specific COVID-precautions (yes vs. no)									
	Coughing or sneezing in the elbow					0.74 (0.57–0.97)	0.027		
	Outcome: Daytime fatigue (reference = no change)								
Change in work situation									
	My work situation has not changed (reference)	-	-	-	-	-	-	-	-
	I was let go	3.51 (1.40–8.82)	0.008						
Occupation									
	Full time job (reference)	-	-	-	-	-	-	-	-
	Part time job			4.52 (2.10–9.69)	<0.001				
Job type									
	Academic (reference)	-	-	-	-	-	-	-	-
	Skilled worker	2.19 (1.29–3.70)	0.004						
	Unskilled worker	2.24 (1.02–4.89)	0.044						
Stamina score (continuous)		0.91 (0.88–0.93)	<0.001	0.95 (0.89–0.96)	<0.001	0.92 (0.89–0.94)	<0.001		
Educational level									
	Elementary school (reference)	-	-	-	-	-	-	-	-
	High school or vocational course							0.37 (0.20–0.69)	0.002
	Short length education	0.40 (0.17–0.95)	0.038					0.39 (0.16–0.92)	0.032
	Medium length education			0.38 (0.16–0.93)	0.034			0.29 (0.14–0.61)	0.001
	Long length education			0.34 (0.13–0.90)	0.031	0.50 (0.25–0.99)	0.046	0.25 (0.10–0.60)	0.002
Smoking during the pandemic (yes vs. no)						1.51 (1.02–2.22)	0.038		
BMI during the pandemic (continuous)				1.05 (1.00–1.11)	0.045				
Specific COVID-precautions (yes vs. no)									
	Using disposable handkerchiefs	1.94 (1.15–3.27)	0.013						
	Limiting use of public transportation					1.48 (1.08–1.99)	0.015		
	Avoid places where many people gather			1.68 (1.04–2.71)	0.034				
	Staying home							1.61 (1.04–2.50)	0.033
Outcome: Restless legs during sleep									
Job type									
	Academic (reference)	-	-	-	-	-	-	-	-
	Administration/Office work					0.49 (0.30–0.78)	0.003		
	Unskilled worker							4.15 (1.33–13.1)	0.014
Stamina score (continuous)		0.97 (0.94–0.99)	0.030	0.93 (0.90–0.98)	0.002	0.94 (0.91–0.97)	<0.001	0.92 (0.88–0.96)	0.001
Educational level									
	Elementary school (reference)	-	-	-	-	-	-	-	-
	Long length education					0.42 (0.18–0.98)	0.045		
Feeling worried about the COVID pandemic and cut-off from social experiences (yes vs. no)								2.16 (1.10–4.23)	0.026
BMI during the pandemic (continuous)		1.05 (1.01–1.09)	0.026	1.10 (1.03–1.18)	0.004	1.07 (1.04–1.09)	<0.001	1.07 (1.02–1.13)	0.009

Analyses were adjusted for age, number of days between answering the questionnaire before the pandemic and the one during, change in work situation during the pandemic, occupation, type of job, stamina score, previous use of anti-depressive medication, cohabitation (living with children and living with another adult), educational level, worry about the pandemic, smoking during the pandemic, body mass index during the pandemic, and COVID-19 precautionary behaviours. OBS: for risk estimates only adjusted for age and time interval between answering the questionnaire before the COVID-19 pandemic and the one after, see Tables S3–S5. Follow-Up Analyses.

## Data Availability

The data underlying this article will be shared on reasonable request to the corresponding author.

## Supplementary Materials

The following are available online at https://www.mdpi.com/article/10.3390/ijerph18147610/s1. Table S1: Political interventions aimed at preventing COVID-19 spread in Denmark; Table S2: Stamina scale; Table S3: Linear regression analyses. Identification of characteristics associated with decrease or increase in mental or physical health characteristics; Table S4: Multinomial logistic regression analyses displaying relative risk ratios (RRR). Identification of characteristics associated with developing a poor quality of sleep phenotype or becoming free of a poor quality of sleep phenotype; Table S5: Linear regression analyses. Identification of characteristics associated with physical and mental health characteristics measured during the COVID-19 pandemic; Table S6: Logistic regression analyses. Identification of characteristics associated with quality of sleep measured during the COVID-19 pandemic.
