# Supplementary material for: Impact of COVID-19 Pandemic on Sleep Quality, Stress Level and Health-Related Quality of Life—A Large Prospective Cohort Study on Adult Danes
Source: Int J Environ Res Public Health. 2021 Jul 17;18(14):7610. doi: 10.3390/ijerph18147610 (PMC8307688; doi:10.3390/ijerph18147610)
Supplement: Supplementary file 1 [file ijerph-18-07610-s001.zip › ijerph-1289310-SI.pdf]

Supplementary material

Impact of COVID-19 pandemic on sleep quality, stress level and health-related quality of life – A large prospective cohort study on adult Danes

Maria Didriksen[1], Thomas Werge[2,3,4], Janna Nissen[1], Michael Schwinn[1], Erik Sørensen[1], Kasper R. Nielsen[5], Mie T. Bruun[6], Karina Banasik[7], Thomas F. Hansen[8], Christian Erikstrup[9], Sisse R. Ostrowski[1], Poul J. Jennum[10,11], Henrik Hjalgrim[12,13], Henrik Ullum[10], Ole B. Pedersen[14]

[1]Department of Clinical Immunology, Copenhagen University Hospital, Rigshospitalet, Copenhagen, Denmark

[2]Institute of Biological Psychiatry, Mental Health Centre Sct. Hans, Mental Health Services, Roskilde, Denmark

[3]Department of Clinical Medicine, University of Copenhagen, Copenhagen, Denmark

[4]The Lundbeck Foundation Initiative for Integrative Psychiatric Research, iPSYCH, Copenhagen, Denmark

[5] Department of Clinical Immunology, Aalborg University Hospital, Aalborg, Denmark

[6]Department of Clinical Immunology, Odense University Hospital, Odense, Denmark

[7]Novo Nordisk Foundation Center for Protein Research, University of Copenhagen, Copenhagen, Denmark

[8]Department of Neurology, Danish Headache Center, Glostrup Research Institute, Copenhagen University Hospital Rigshospitalet-Glostrup, Glostrup, Denmark

[9]Department of Clinical Immunology, Aarhus University Hospital, Aarhus, Denmark

[10]Department of Clinical Neurophysiology, Danish Center for Sleep Medicine, Copenhagen University Hospital, Rigshospitalet, Glostrup, Denmark

[12]Faculty of Health, University of Copenhagen, Copenhagen, Denmark

[12] Department of Epidemiology Research, Statens Serum Institut, Copenhagen, Denmark

[13]Department of Hematology, Copenhagen University Hospital, Rigshospitalet, Copenhagen, Denmark

[14] Department of Clinical Immunology, Zealand University Hospital, Køge, Denmark

Table S1: Political interventions aimed at preventing COVID-19 spread in Denmark

**Date**	**Intervention**
2020-02-06	Testing strategy towards risk of transmission
2020-02-26	First confirmed COVID-19 case in Denmark
2020-03-03	Home-quarantine for 14 days in case of exposure to COVID-19 or travelling to high-risk areas
2020-03-06	Gatherings of 1,000 individuals were recommended to be postponed or cancelled
2020-03-11	Government employees without critical function were sent home, private sector employees were recommended to work from home, schools and childcare facilities were closed except for children of parents serving critical functions, and gatherings of more than 100 were prohibited. Testing strategy was changed to only include individuals with severe symptoms, vulnerable individuals or individuals with critical functions.
2020-03-14	Closing of borders
2020-03-17	Selected workplaces with close human contact were closed like sports facilities, gyms, shopping centres, hairdressers and others. Gatherings of more than 10 individuals were prohibited
2020-04-15	Reopening of the society starting with primarily schools and childcare up to grade 5. All reopening initiatives were conditioned on the ability to introduce preventive measures minimizing close contact and securing hygiene.
2020-04-23	Reopening of small businesses with close contact like e.g. hairdressers, driving schools, and physiotherapists.
2020-05-11	All shops were reopened.
2020-05-12	Testing strategy broadened out to include testing of individuals at risk of COVID-19, those with symptoms of COVID-19 disease, follow up after COVID-19 symptoms as well as a general SARS-CoV-2 surveillance among health care workers and the general population.
2020-05-18	Reopening of school grades 6 to 10, outdoor sports activities, as well as cafés and restaurants
2020-05-20	Reopening of teaching institutions, museums, theatres, and outdoor zoo’s
2020-06-08	Reopening of borders to selected countries, allowing gatherings of up to 50 individuals, selected indoor sports activities, and workers to return to government institutions

Table S2: Stamina scale

**Item**	
1	I believe I can achieve my goals, even if there are obstacles
2	Under pressure, I stay focused and think clearly
3	I am not easily discouraged by failure
4	I am able to adapt when changes occur
5	I can deal with whatever comes my way
6	I try to see the humorous side of things when I am faced with problems
7	Having to cope with stress can make me stronger
8	I tend to bounce back after illness, injury or other hardships
9	I think of myself as a strong person when dealing with life's challenges and difficulties
**Possible answers:**	0= "not true at all", 1= "rarely true", 2= "sometimes true", 3= "often true", and 4= "true nearly all the time"

Table S3. Linear regression analyses. Identification of characteristics associated with decrease or increase in mental or physical health characteristics

	Men (N =12,150)		Women (N =14,303)	
	Coef. (95% CI)	P	Coef. (95% CI)	P
Outcome: Change in mental component score[×]				
Change in work situation				
My work situation has not changed (reference)	-	-	-	-
**I work/study from home**	**-0.924 (-1.263- -0.585)**	**0.004***	**-0.455 (-0.799-0.032)**	**0.015**
I was sent home without being able to work	-0.521 (-1.328-0.286)	0.206	0.403 (-0.483-1.289)	0.372
I have taken a vacation or a leave of absence	-0.261 (-2.153-1.631)	0.177	0.402 (-2.193-2.997)	0.762
**I was let go**	**-1.736 (-2.963- -0.508)**	**0.006***	**-1.919 (-3.353-0.485)**	**0.009***
**I still go in to work, but my work situation has changed**	**-0.566 (-0.955- -0.177)**	**0.004***	-0.385 (-0.799-0.032)	0.070
Occupation				
Full time job (reference)	-	-	-	-
Part time job	0.005 (-0.759-0.769)	0.989	-0.366 (-0.805-0.073)	0.102
Self employed	0.056 (-0.528-0.640)	0.850	-0.024 (-0.877-0.829)	0.956
Student	**-2.138 (-2.181- -1.458)**	**<0.001**	**-2.329 (-2.289- -1.764)**	**<0.001**
Stay-at-home by choice	-1.651 (-4.487-1.185)	0.254	0.809 (-0.862-2.481)	0.342
Unemployed	-0.153 (-1.077-0.770)	0.745	0.617 (-0.293-1.156)	0.184
Long-term sick leave/parental leave	**-3.942 (-5.796- -2.088)**	**<0.001**	-0.908 (-1.910-0.094)	0.076
Retired	-0.022 (-0.471-0.428)	0.924	**-1.207 (-1.734-0.680)**	**<0.001**
Other	1.034 (-0.213-2.291)	0.104	**-1.091 (-2.045- -0.137)**	**0.025**
Type of job				
Academic (reference)	-	-	-	-
Administration/Office work	-0.103 (-0.859-0.654)	0.790	0.384 (-0.644-1.411)	0.464
Sales/Service	-0.234 (-1.104-0.637)	0.599	0.139 (-1.893-1.170)	0.792
Skilled worker	-0.088 (-0.838-0.661)	0.818	0.735 (-0.103-1.572)	0.086
Unskilled worker	0.009 (-0.709-0.729)	0.979	0.221 (-0.606-1.049)	0.600
Other	0.054 (-0.766-0.873)	0.898	-0.039 (-0.949-0.872)	0.934
**Stamina score**	**0.143 (0.119-0.167)**	**<0.001***	**0.161 (0.137-0.186)**	**<0.001***
**Ever been prescribed anti-depressive medication**	0.015 (-0.456-0.487)	0.949*	0.313 (-0.104-0.730)	0.141*
Living with child(ren)				
No (reference)	-	-	-	-
Below school age (<5 years old)	-0.306 (-1.437-0.826)	0.596	0.020 (-1.216-1.256)	0.975
School age (5 – 18 years old)	0.241 (-0.044-0.527)	0.098*	0.298 (-0.006-0.603)	0.055
Living with another adult (yes vs. no)	-0.281 (-0.565- -0.003)	0.052*	0.251 (-0.050-0.551)	0.102

Educational level				
Elementary school (reference)	-	-	-	-
High school or vocational course	0.199 (-0.260-0.659)	0.395	0.334 (-0.163-0.831)	0.188
Short length education	0.482 (-0.118-1.082)	0.115	**0.891 (0.182-1.599)**	**0.014**
Medium length education	0.244 (-0.273-0.759)	0.355	0.332 (-0.118-0.852)	0.210
**Long length education**	0.488 (-0.039-1.015)	0.070	**0.995 (0.395-1.598)**	**<0.001**
**Feeling worried about the COVID pandemic and cut-off from social experiences (most of the time vs. never or sometime)**	**0.506 (0.126-0.885)**	**0.009***	0.444 (-0.019-0.906)	0.060
Daily alcohol intake during the pandemic (yes vs. no)	-0.340 (-0.865-0.185)	0.204	-0.409 (-1.182-0.364)	0.299
Smoking during the pandemic (yes vs. no)	-0.333 (-0.745 -0.079)	0.113	-0.229 (-0.665-0.205)	0.301*
**BMI during the pandemic (continuous)**	-0.019 (-0.054-0.015)	0.269	**0.034 (0.005-0.063)**	**0.022***
COVID-19 precautionary behavior (yes vs. no)				
**Frequently washing hands**	**-0.485 (-0.922-0.048)**	**0.030**	-0.365 (-0.846-0.116)	0.137
Coughing or sneezing in the elbow	-0.174 (-0.478-0.131)	0.264	-0.279 (-0.643-0.083)	0.131
**Using disposable handkerchiefs**	**-0.581 (-0.984-0.179)**	**0.005**	**-0.411 (-0.763-0.058)**	**0.022**
**Wearing disposable masks**	**-1.590 (-2.698-0.483)**	**0.005***	-0.663 (-1.602-0.278)	0.167
Avoiding handshakes	-0.270 (-0.722-0.182)	0.241	0.175 (-0.336-0.686)	0.503
Stopped greeting people with hugs and/or kisses on both cheeks	-0.129 (-0.453-0.196)	0.437	-0.251 (-0.643-0.140)	0.209
**Limiting use of public transportation**	**-0.409 (-0.675- -0.142)**	**0.003**	**-0.628 (-0.910- -0.345)**	**<0.001**
Avoid places where many people gather	-0.237 (-0.495-0.021)	0.072	**-0.457 (-0.761-0.153)**	**0.003**
Staying home	**-0.839 (-1.114- -0.563)**	**<0.001**	**-0.498 (-0.786-0.210)**	**0.001**
Working from home more often	**-0.314 (-0.582-0.046)**	**0.022**	-0.016 (-0.309-0.278)	0.916
**Avoiding travels outside one's own country or region**	**-0.397 (-0.649- -0.144)**	**0.002***	-0.208 (-0.492-0.075)	0.150
Outcome: Change in physical component score[×]				
Change in work situation				
My work situation has not changed (reference)	-	-	-	-
I work/study from home	0.156 (-0.092-0.405)	0.217	-0.053 (-0.303-0.197)	0.678
I was sent home without being able to work	0.216 (-0.375-0.807)	0.474	0.109 (-0.499-0.716)	0.726
I have taken a vacation or a leave of absence	-0.083 (-1.469-1.304)	0.907	-0.819 (-2.598-0.958)	0.366
**I was let go**	0.574 (-0.326-1.473)	0.211	**0.969 (-0.014-0.1951)**	**0.053***
I still go in to work, but my work situation has changed	-0.094 (-0.379-0.191)	0.520	-0.168 (-0.453-0.116)	0.247
Occupation				
Full time job (reference)	-	-	-	-
Part time job	-0.333 (-0.919-0.254)	0.266	-0.169 (-0.486-0.146)	0.293
Self employed	0.048 (-0.401-0.496)	0.835	0.563 (-0.052-1.178)	0.073
Student	0.267 (-0.255-0.788)	0.317	0.329 (-0.079-0.737	0.114
Stay-at-home by choice	1.545 (-0.631-3.721)	0.164	-0.215 (-1.419-0.989)	0.726
Unemployed	0.054 (-0.655-0.763)	0.882	-0.221 (-0.877-0.435)	0.509

**Long-term sick leave/parental leave**	-5.710 (-7.133- -4.288)	<0.001	-1.491 (-2.214- -0.769)	<0.001
**Retired**	-0.571 (-0.916- -0.226)	0.001	-0.429 (-0.809- -0.050)	0.026
Other	-0.423 (-1.384-0.538)	0388	0.408 (-0.279-1.095)	0.245
Job type				
Academic (reference)	-	-	-	-
Administration/Office work	0.014 (-0.275-0.304)	0.922	-0.055 (-0.318-0.209)	0.683
**Sales/Service**	**-0.548 (-0.991- -0.106)**	**0.015***	0.386 (-0.118-0.892)	0.134
Skilled worker	-0.078 (-0.379-0.223)	0.612	-0.068 (-0.573-0.436)	0.790
**Unskilled worker**	-0.350 (-0.896-0.195)	0.208	**-0.599 (-1.197- -0.002)**	**0.049***
Other	-0.014 (-0.399-0.372)	0.945	-0.268 (-0.638-0.101)	0.155
**Stamina score**	0.011 (-0.008-0.029)	0.245	**-0.002 (-0.020-0.015)**	**0.786***
Ever been prescribed anti-depressive medication	-0.570 (-0.932- -0.208)	0.002	-0.239 (-0.539-0.060)	0.117
Living with child(ren)				
No (reference)	-	-	-	-
Below school age (<5 years old)	0.216 (-0.652-1.085)	0.626	-0.275 (-1.163-0.613)	0.544
School age (5 – 18 years old)	0.155 (-0.064-0.375)	0.165	-0.040 (-0.259-0.179)	0.720
Living with another adult (yes vs. no)	0.167 (-0.053-0.387)	0.138	-0.067 (-0.284-0.151)	0.548
Educational level				
Elementary school (reference)	-	-	-	-
High school or vocational course	0.107 (-0.248-0.461)	0.556	-0.238 (-0.600-0.125)	0.199
Short length education	0.095 (-0.367-0.559)	0.685	-0.379 (-0.896-0.137)	0.150
Medium length education	0.150 (-0.248-0.548)	0.460	-0.003 (-0.382-0.376)	0.988
**Long length education**	0.303 (-0.104-0.709)	0.144	-0.319 (-0.758-0.121)	0.155
Feeling worried about the COVID pandemic and cut-off from social experiences (most of the time vs. never or sometime)	0.431 (0.140-0.722)	0.004	**0.105 (-0.228-0.437)**	**0.538***
Daily alcohol intake during the pandemic (yes vs. no)	-0.185 (-0.589-0.219)	0.370	-0.141 (-0.691-0.409)	0.615
Smoking during the pandemic (yes vs. no)	0.199 (-0.117-0.515)	0.218	0.029 (-0.283-0.342)	0.853
**BMI during the pandemic (continuous)**	**-0.039 (-0.065- -0.013)**	**0.004***	**-0.044 (-0.065- -0.023)**	**<0.001***
Specific COVID-precautions (yes vs. no)				
Frequently washing hands	0.112 (-0.224-0.448)	0.512	-0.001 (-0.347-0.345)	0.996
Coughing or sneezing in the elbow	0.139 (-0.094-0.374)	0.243	0.229 (-0.038-0.484)	0.094
**Using disposable handkerchiefs**	**-0.235 (-0.544-0.074)**	**0.135***	-0.013 (-0.267-0.240)	0.919
**Wearing disposable masks**	-0.680 (-1.530-0.169)	0.117	**-0.625 (-1.301-0.051)**	**0.070***
Avoiding handshakes	-0.074 (-0.421-0.273)	0.677	-0.098 (-0.465-0.269)	0.601
Stopped greeting people with hugs and/or kisses on both cheeks	-0.042 (-0.291-0.207)	0.740	0.051 (-0.231-0.332)	0.725
**Limiting use of public transportation**	-0.080 (-0.285-0.124)	0.442	**0.053 (-0.150-0.256)**	**0.610***
Avoid places where many people gather	0.017 (-0.181-0.215)	0.865	-0.130 (-0.349-0.089)	0.243
**Staying home**	**-0.267 (-0.479- -0.556)**	**0.013***	**-0.471 (-0.678- -0.263)**	**<0.001***
Working from home more often	0.083 (-0.123-0.289)	0.428	-0.033 (-0.244-0.178)	0.761

Avoiding travels outside one's own country or region	0.040 (-0.153-0.234)	0.683	0.036 (-0.168-0.239)	0.731
**Outcome: Change in stress score**[×]				
	Men (N=5,790)		Women (N=6.617)	
Change in work situation				
My work situation has not changed (reference)	-	-	-	-
I work/study from home	0.421 (0.104-0.737)	0.009	0.289 (-0.056-0.635)	0.101
I was sent home without being able to work	-0.175 (-0.917-0.567)	0.644	-0.108 (-0.931-0.715)	0.797
I have taken a vacation or a leave of absence	1.685 (-0.238-3.607)	0.086	1.554 (-1.051-4.159)	0.242
I was let go	1.574 (0.415-2.732)	0.008	1.719 (0.437-3.001)	0.009
I still go in to work, but my work situation has changed	0.426 (0.058-0.794)	0.023	0.417 (0.022-0.812)	0.038
**Stamina score (continuous)**	**-0.116 (-0.141- -0.091)**	**<0.001***	**-0.147 (-0.172- -0.121)**	**<0.001***
Occupation				
Full time job (reference)	-	-	-	-
Part time job	0.405 (-0.454-1.264)	0.355	0.148 (-0.288-0.585)	0.505
Self employed	0.333 (-0.247-0.914)	0.260	0.152 (-0.699-1.003)	0.727
Student	0.822 (0.233-1.413)	0.006	1.771 (1.273-2.268)	<0.001
Stay-at-home by choice	1.269 (-1.415-3.953)	0.354	0.012 (-1.559-1.584)	0.988
Unemployed	1.362 (0.454-2.271)	0.003	0.149 (-0.750-1.047)	0.746
Long-term sick leave/parental leave	3.643 (1.579-5.708)	0.001	-0.604 (-1.842-0.634)	0.339
Retired	0.431 (-0.213-1.076)	0.190	0.085 (-0.608-0.778)	0.809
Other	0.027 (-1.391-1.446)	0.969	-0.412 (-1.433-0.609)	0.429
Job type				
Academic (reference)	-	-	-	-
**Administration/Office work**	**-0.365 (-0.723- -0.006)**	**0.046***	**-0.719 (-1.074- -0.365)**	**<0.001***
**Sales/Service**	**-0.749 (-1.299- -0.199)**	**0.008***	-0.273 (-0.918-0.373)	0.406
Skilled worker	-0.431 (-0.809- -0.052)	0.026	-0.388 (-1.058-0.282)	0.256
Unskilled worker	-0.219 (-0.931-0.494)	0.547	-0.484 (-1.292-0.325)	0.241
Other	-0.410 (-0.908-0.087)	0.106	-0.089 (-0.582-0.402)	0.723
Ever been prescribed anti-depressive medication	0.147 (-0.372-0.666)	0.579	0.312 (-0.132-0.755)	0.168
Living with child(ren)				
No (reference)	-	-	-	-
Below school age (<5 years old)	0.037 (-1.039-1.113)	0.946	0.689 (-0.824-2.203)	0.372
School age (5 – 18 years old)	-0.146 (-0.434-0.142)	0.322	-0.208 (-0.505-0.089)	0.170
Living with another adult (yes vs. no)	0.189 (-0.108-0.487)	0.212	0.119 (-0.187-0.426)	0.445
Educational level				
Elementary school (reference)	-	-	-	-
High school or vocational course	-0.125 (-0.618-0.368)	0.618	0.018 (-0.482-0.518)	0.943
Short length education	-0.139 (-0.767-0.488)	0.664	-0.204 (-0.930-0.522)	0.582

Medium length education	0.412 (-0.138-0.962)	0.142	0.309 (-0.225-0.840)	0.257
Long length education	0.113 (-0.449-0.674)	0.693	0.506 (-0.112-1.122)	0.108
Feeling worried about the COVID pandemic and cut-off from social experiences (most of the time vs. never or sometime)	-0.047 (-0.429-0.336)	0.811	0.087 (-0.377-0.551)	0.713
Daily alcohol intake during the pandemic (yes vs. no)	-0.055 (-0.755-0.644)	0.877	-0.143 (-1.09-0.805)	0.767
Smoking during the pandemic (yes vs. no)	0.002 (-0.425-0.429)	0.994	0.036 (-0.398-0.470)	0.871
BMI during the pandemic (continuous)	-0.013 (-0.048-0.022)	0.458	-0.014 (-0.043-0.015)	0.329
Specific COVID-precautions (yes vs. no)				
Frequently washing hands	0.139 (-0.284-0.563)	0.518	0.022 (-0.449-0.493)	0.929
Coughing or sneezing in the elbow	0.018 (-0.289-0.326)	0.904	-0.034 (-0.396-0.328)	0.854
Using disposable handkerchiefs	-0.001 (-0.467-0.464)	0.996	0.021 (-0.350-0.392)	0.912
Wearing disposable masks	0.447 (-0.725-1.619)	0.454	0.371 (-0.614-0.1357)	0.460
Avoiding handshakes	0.161 (-0.284-0.607)	0.478	0.171 (-0.309-0.652)	0.485
Stopped greeting people with hugs and/or kisses on both cheeks	-0.028 (-0.355-0.300)	0.869	0.292 (-0.086-0.669)	0.130
Limiting use of public transportation	0.186 (-0.091-0.463)	0.188	0.431 (0.143-0.718)	0.003
Avoid places where many people gather	0.057 (-0.203-0.318)	0.667	0.309 (0.011-0.609)	0.042
Staying home	0.349 (0.063-0.634)	0.017	0.506 (0.213-0.799)	0.001
Working from home more often	0.183 (-0.078-0.444)	0.170	0.129 (-0.158-0.416)	0.377
Avoiding travels outside one’s own country or region	0.048 (-0.209-0.305)	0.715	0.194 (-0.089-0.478)	0.180

[*] The presented coefficients are crude effect estimates only adjusted for age and number of days between answering the first questionnaire and the questionnaire answered during the COVID-19 pandemic. *indicates the covariates with a P-value<0.05 in the model comprising all covariates.

×changes in scores were calculated by subtracting the score before the COVID-19 pandemic from the score during. A negative score represents a decrease, while a positive score represents an increase

Factors with a P-value<0.05 are marked in bold writing

Table S4. Multinomial logistic regression analyses displaying relative risk ratios (RRR). Identification of characteristics associated with developing a poor quality of sleep phenotype or becoming free of a poor quality of sleep phenotype

	Men (N =5,790)				Women (N =6,617)			
	From non-case to case		From case to non-case		From non-case to case		From case to non-case	
	RRR (95% CI)	P	RRR (95% CI)	P	RRR (95% CI)	P	RRR (95% CI)	P
Outcome: Insomnia (no change = reference)								
Change in work situation								
My work situation has not changed (reference)	-	-	-	-	-	-	-	-
I work/study from home	0.98 (0.79-1.20)	0.822	0.97 (0.78-1.20)	0.751	1.15 (0.96-1.37)	0.125	0.96 (0.78-1.18)	0.720
I was sent home without being able to work	1.09 (0.68-1.75)	0.729	1.14 (0.71-1.84)	0.591	1.43 (0.96-2.12)	0.076	1.15 (0.72-1.84)	0.555
I have taken a vacation or a leave of absence	1.55 (0.52-4.61)	0.433	1.19 (0.35-4.08)	0.778	0.87 (0.20-3.84)	0.857	0.43 (0.06-3.29)	0.418
I was let go	1.10 (0.52-2.35)	0.800	1.58 (0.81-3.06)	0.180	1.23 (0.67-2.28)	0.502	0.49 (0.18-1.37)	0.175
I still go in to work, but my work situation has changed	0.88 (0.69-1.13)	0.322	1.05 (0.83-1.34)	0.682	1.17 (0.95-1.44)	0.142	1.09 (0.87-1.36)	0.443
Occupation								
Full time job (reference)	-	-	-	-	-	-	-	-
Part time job	0.98 (0.54-1.76)	0.945	1.64 (1.02-2.63)	0.042	1.02 (0.80-1.28)	0.920	0.96 (0.75-1.239	0.731
Self employed	0.84 (0.56-1.26)	0.405	1.02 (0.71-1.48)	0.911	0.83 (0.50-1.37)	0.460	1.20 (0.77-1.86)	0.424
Student	1.29 (0.89-1.87)	0.176	1.04 (0.68-1.59)	0.861	1.31 (1.04-1.65)	0.022	1.13 (0.82-1.57)	0.459
Stay-at-home by choice	Too few		1.14 (0.25-5.20)	0.863	1.48 (0.68-3.25)	0.324	2.40 (1.19-4.84)	0.014
Unemployed	2.16 (1.34-3.48)	0.002	1.46 (0.83-2.56)	0.190	1.10 (0.70-1.73)	0.682	1.28 (0.77-2.10)	0.339
Long-term sick leave/parental leave	4.15 (1,73-9.94)	0.001	Too few		1.74 (1.01-3.01)	0.047	1.69 (0.87-3.28)	0.121
Retired	1.16 (0.79-1.72)	0.446	0.85 (0.57-1.29)	0.446	1.06 (0.70-1.60)	0.779	1.16 (0.83-1.62)	0.376
Other	1.33 (0.59-3.02)	0.488	0.72 (0.25-2.04)	0.536	0.79 (0.43-1.45)	0.439	0.86 (0.49-1.53)	0.613
Job type								
Academic (reference)	-	-	-	-	-	-	-	-
Administration/Office work	0.92 (0.72-1.17)	0.494	0.86 (0.67-1.10)	0.223	0.99 (0.81-1.20)	0.885	1.01 (0.82-1.24)	0.944
Sales/Service	1.16 (0.81-1.65)	0.424	1.05 (0.73-1.50)	0.799	0.84 (0.58-1.21)	0.340	0.79 (0.52-1.20)	0.272
Skilled worker	1.09 (0.85-1.40)	0.491	0.92 (0.72-1.19)	0.540	1.02 (0.71-1.48)	0.900	1.08 (0.74-1.58)	0.686
**Unskilled worker**	**1.64 (1.09-2.46)**	**0.017***	0.81 (0.48-1.35)	0.411	0.98 (0.63-1.52)	0.916	0.73 (0.43-1.23)	0.235
Other	0.94 (0.67-1.33)	0.731	1.11 (0.81-1.52)	0.512	1.07 (0.82-1.40)	0.623	1.27 (0.97-1.66)	0.083
**Stamina score (continuous)**	**0.95 (0.94-0.97)**	**<0.001***	0.98 (0.97-1.00)	0.052	**0.96 (0.94-0.97)**	**<0.001***	**1.00 (0.99-1.02)**	**0.547***
**Ever been prescribed anti-depressive medication**	1.48 (1.10-1.99)	0.010	1.01 (0.72-1.43)	0.935	**1.48 (1.18-1.82)**	**<0.001***	1.23 (0.97-1.56)	0.091
Living with child(ren)								
No (reference)	-	-	-	-	-	-	-	-
Below school age (<5 years old)	1.08 (0.56-2.10)	0.809	1.33 (0.70-2.53)	0.377	0.97 (0.48-1.96)	0.933	0.49 (0.15-1.60)	0.239
**School age (5 – 18 years old)**	0.79 (0.66-0.95)	0.11	**0.73 (0.61-0.87)**	**0.001***	0.83 (0.71-0.96)	0.015	**0.75 (0.64-0.89)**	**0.001***
Living with another adult (yes vs. no)	0.82 (0.68-0.99)	0.036	0.89 (0.73-1.08)	0.246	0.90 (0.77-1.05)	0.190	0.97 (0.82-1.16)	0.756
Educational level								
Elementary school (reference)	-	-	-	-	-	-	-	-
High school or vocational course	0.73 (0.54-0.99)	0.041	1.10 (0.78-1.55)	0.597	0.94 (0.73-1.21)	0.641	0.82 (0.62-1.10)	0.187
Short length education	0.72 (0.49-1.08)	0.112	1.11 (0.73-1.70)	0.635	0.69 (0.46-1.05)	0.086	0.96 (0.66-1.42)	0.851

Medium length education	0.77 (0.55-1.08)	0.128	1.09 (0.74-1.60)	0.669	0.78 (0.59-1.03)	0.081	0.75 (0.56-1.01)	0.055
**Long length education**	0.88 (0.62-1.24)	0.464	**1.47 (1.01-2.15)**	**0.045***	0.87 (0.63-1.20)	0.394	0.87 (0.62-1.23)	0.436
Feeling worried about the COVID pandemic and cut-off from social experiences (yes vs. no)	0.70 (0.54-0.92)	0.011	0.87 (0.68-1.13)	0.297	0.98 (0.76-1.25)	0.843	0.90 (0.69-1.17)	0.425
Daily alcohol intake during the pandemic (yes vs. no)	1.34 (0.89-2.01)	0.161	0.88 (0.55-1.40)	0.582	0.90 (0.50-1.63)	0.733	1.39 (0.89-2.15)	0.145
Smoking during the pandemic (yes vs. no)	1.20 (0.92-1.56)	0.171	0.95 (0.71-1.26)	0.702	1.07 (0.86-1.33)	0.537	0.92 (0.71-1.19)	0.526
BMI during the pandemic (continuous)	1.01 (0.99-1.03)	0.422	1.01 (0.99-1.03)	0.406	1.01 (1.00-1.03)	0.116	1.00 (0.99-1.02)	0.828
Specific COVID-precautions (yes vs. no)								
Frequently washing hands	1.09 (0.81-1.44)	0.560	0.97 (0.73-1.28)	0.816	1.19 (0.93-1.53)	0.170	1.22 (0.91-1.63)	0.183
**Coughing or sneezing in the elbow**	0.97 (0.80-1.18)	0.782	0.99 (0.81-1.21)	0.898	**0.92 (0.77-1.10)**	**0.347***	1.13 (0.91-1.41)	0.257
Using disposable handkerchiefs	1.22 (0.91-1.61)	0.180	1.20 (0.91-1.60)	0.200	1.06 (0.87-1.29)	0.584	0.95 (0.78-1.16)	0.624
Wearing disposable masks	1.10 (0.52-2.34)	0.797	1.61 (0.83-3.12)	0.156	0.96 (0.57-1.62)	0.876	0.91 (0.51-1.61)	0.755
Avoiding handshakes	0.84 (0.64-1.11)	0.228	0.98 (0.73-1.32)	0.908	0.94 (0.74-1.19)	0.604	1.08 (0.80-1.44)	0.618
Stopped greeting people with hugs and/or kisses on both cheeks	1.03 (0.83-1.27)	0.807	0.94 (0.76-1.16)	0.558	0.98 (0.81-1.18)	0.816	1.15 (0.91-1.45)	0.228
Limiting use of public transportation	1.07 (0.90-1.27)	0.462	1.04 (0.87-1.25)	0.643	1.08 (0.94-1.26)	0.270	1.16 (0.98-1.36)	0.081
Avoid places where many people gather	0.99 (0.85-1.18)	0.996	0.84 (0.72-1.00)	0.056	1.05 (0.90-1.23)	0.510	1.13 (0.95-1.35)	0.152
Staying home	0.94 (0.78-1.14)	0.538	0.91 (0.75-1.10)	0.332	1.19 (1.03-1.38)	0.020	1.16 (0.98-1.37)	0.083
Working from home more often	0.98 (0.83-1.16)	0.802	1.05 (0.88-1.24)	0.608	1.01 (0.87-1.17)	0.916	0.95 (0.81-1.12)	0.560
Avoiding travels outside one's own country or region	0.94 (0.80-1.11)	0.465	0.99 (0.84-1.17)	0.906	0.94 (0.81-1.09)	0.418	1.16 (0.98-1.37)	0.077

Outcome: Daytime fatigue (reference = no change)

Change in work situation								
My work situation has not changed (reference)	-	-	-	-	-	-	-	-
I work/study from home	1.02 (0.75-1.40)	0.882	0.87 (0.58-1.31)	0.505	1.01 (0.80-1.29)	0.924	1.06 (0.75-1.50)	0.725
I was sent home without being able to work	1.25 (9.64-2.44	0.512	1.19 (0.51-2.79)	0.684	1.58 (0.97-2.58)	0.067	0.68 (0.25-1.88)	0.455
I have taken a vacation or a leave of absence	1.93 (0.45-8.33)	0.379	1.43 (0.19-10.7)	0.731	Too few		1.85 (0.24-14.1)	0.552
**I was let go**	**2.38 (1.06-5.33)**	**0.036***	1.07 (0.26-4.47)	0.926	2.22 (1.13-4.36)	0.021	2.33 (0.91-5.96)	0.079
I still go in to work, but my work situation has changed	1.05 (0.73-1.51)	0.795	0.69 (0.41-1.16)	0.163	1.09 (0.82-1.45)	0.551	0.92 (0.60-1.40)	0.690
Occupation								
Full time job (reference)	-	-	-	-	-	-	-	-
**Part time job**	1.06 (0.46-2.43)	0.898	**3.24 (1.65-6.38)**	**0.001***	0.94 (0.67-1.32)	0.728	1.16 (0.75-1.79)	0.495
Self employed	1.13 (0.65-1.97)	0.665	1.47 (0.76-2.85)	0.252	1.16 (0.62-2.17)	0.644	0.58 (0.18-1.85)	0.358
Student	0.99 (0.60-1.62)	0.956	0.74 (0.35-1.56)	0.427	1.45 (1.10-1.92)	0.009	1.25 (0.79-1.98)	0.349
Stay-at-home by choice	7.14 (1.92-26.5)	0.003	Too few		0.96 (0.29-3.13)	0.946	Too few	
Unemployed	2.19 (1.17-4.07)	0.014	1.32 (0.47-3.67)	0.597	2.46 (1.56-3.88)	<0.001	2.07 (1.02-4.18)	0.044
Long-term sick leave/parental leave	6.15 (2.22-17.0)	<0.001	2.30 (0.30-17.5)	0.423	2.29 (1.21-4.35)	<0.001	3.16 (1.40-7.10)	0.005
Retired	1.78 (0.97-3.27)	0.060	4.94 (2.82-8.69)	<0.001	2.81 (1.75-4.50)	<0.001	1.07 (0.49-2.31)	0.868
Other	0.52 (0.07-3.79)	0.517	1.92 (0.56-8.07)	0.371	1.29 (0.62-2.69)	0.504	1.49 (0.60-3.78)	0.391
Job type								
Academic (reference)	-	-	-	-	-	-	-	-
Administration/Office work	0.90 (0.60-1.32)	0.567	1.20 (0.72-1.98)	0.490	0.82 (0.61-1.10)	0.192	1.50 (1.01-2.22)	0.045
Sales/Service	0.66 (0.33-1.32)	0.236	1.61 (0.82-3.16)	0.169	1.40 (0.91-2.15)	0.124	1.71 (0.89-3.25)	0.102
**Skilled worker**	**2.02 (1.44-2.83)**	**<0.001***	2.38 (1.52-3.72)	<0.001	1.22 (0.74-2.02)	0.432	2.63 (1.47-4.71)	0.001

**Unskilled worker**	**2.41 (1.37-4.22)**	**0.002***	2.38 (1.09-5.17)	0.029	2.31 (1.43-3.73)	0.001	2.10 (0.98-4.52)	0.057
Other	1.21 (0.73-1.99)	0.463	1.09 (0.52-2.26)	0.824	1.11 (0.76-1.63)	0.576	1.83 (1.11-3.02)	0.017
**Stamina score (continuous)**	**0.90 (0.89-0.92)**	**<0.001***	**0.93 (0.91-0.96)**	**<0.001***	**0.91 (0.90-0.92)**	**<0.001***	0.95 (0.92-0.97)	<0.001
Ever been prescribed anti-depressive medication (yes vs. no)	2.69 (1.88-3.86)	<0.001	1.20 (0.66-2.18)	0.557	1.87 (1.44-2.44)	<0.001	1.91 (1.32-2.77)	0.001
Living with child(ren)								
No (reference)	-	-	-	-	-	-	-	-
Below school age (<5 years old)	0.90 (0.35-2.29)	0.825	1.83 (0.63-5.27)	0.263	0.87 (0.34-2.21)	0.768	1.62 (0.49-5.35)	0.427
School age (5 – 18 years old)	0.83 (0.64-1.08)	0.176	1.15 (0.80-1.64)	0.455	0.84 (0.69-1.03)	0.091	1.10 (0.81-1.48)	0.553
Living with another adult (yes vs. no)	0.91 (0.69-1.20)	0.492	1.23 (0.84-1.81)	0.281	0.79 (0.64-0.96)	0.020	0.96 (0.71-1.31)	0.800
Educational level								
Elementary school (reference)	-	-	-	-	-	-	-	-
**High school or vocational course**	0.75 (0.50-1.12)	0.162	0.59 (0.37-0.92)	0.023	0.74 (0.54-1.00)	0.049	**0.65 (0.43-1.00)**	**0.050***
**Short length education**	**0.34 (0.17-0.67)**	**0.002***	0.46 (0.24-0.91)	0.025	0.52 (0.29-0.91)	0.022	**0.55 (0.27-1.12)**	**0.100***
**Medium length education**	0.52 (0.32-0.85)	0.009	**0.29 (0.16-0.55)**	**<0.001***	0.65 (0.46-0.92)	0.016	**0.42 (0.26-0.70)**	**0.001***
**Long length education**	0.60 (0.37-0.97)	0.039	**0.25 (0.13-0.50)**	**<0.001***	**0.51 (0.22-0.80)**	**0.003***	**0.46 (0.15-0.84)**	**0.012***
Feeling worried about the COVID pandemic and cut-off from social experiences (yes vs. no)	0.75 (0.50-1.12)	0.156	0.60 (0.34-1.04)	0.068	0.96 (0.68-1.35)	0.821	0.84 (0.51-1.40)	0.504
Daily alcohol intake during the pandemic (yes vs. no)	1.27 (0.66-2.46)	0.477	0.87 (0.35-2.16)	0.756	1.00 (0.43-2.31)	0.998	2.36 (1.11-4.98)	0.025
**Smoking during the pandemic (yes vs. no)**	1.47 (1.04-2.09)	0.031	1.19 (0.73-1.94)	0.482	**1.46 (1.13-1.90)**	**0.004***	1.55 (1.06-2.25)	0.023
**BMI during the pandemic (continuous)**	1.03 (0.99-1.06)	0.103	**1.04 (0.99-1.08)**	**0.079***	1.03 (1.01-1.05)	0.001	1.02 (0.99-1.05)	0.099
Specific COVID-precautions (yes vs. no)								
Frequently washing hands	1.12 (0.75-1.68)	0.577	0.95 (0.58-1.56)	0.834	0.94 (0.70-1.27)	0.703	0.73 (0.48-1.11)	0.140
Coughing or sneezing in the elbow	0.91 (0.69-1.20)	0.506	1.37 (0.91-2.05)	0.130	0.95 (0.75-1.20)	0.669	0.94 (0.66-1.34)	0.744
**Using disposable handkerchiefs**	**1.40 (0.93-2.12)**	**0.105***	1.08 (0.62-1.87)	0.790	1.03 (0.78-1.35)	0.841	1.31 (0.91-1.86)	0.142
Wearing disposable masks	1.14 (0.41-3.17)	0.798	0.50 (0.07-3.61)	0.489	1.49 (0.83-2.68)	0.184	0.75 (0.23-2.37)	0.618
Avoiding handshakes	0.95 (0.63-1.43)	0.807	0.88 (0.52-1.46)	0.611	0.92 (0.68-1.24)	0.593	0.91 (0.58-1.43)	0.673
Stopped greeting people with hugs and/or kisses on both cheeks	1.19 (0.87-1.63)	0.284	1.01 (0.68-1.51)	0.944	0.90 (0.71-1.15)	0.407	0.90 (0.63-1.28)	0.551
**Limiting use of public transportation**	1.14 (0.88-1.47)	0.303	1.22 (0.88-1.69)	0.224	**1.43 (1.19-1.73)**	**<0.001***	1.04 (0.78-1.38)	0.813
**Avoid places where many people gather**	1.12 (0.87-1.43)	0.380	**1.30 (0.94-1.80)**	**0.112***	1.21 (0.98-1.49)	0.078	1.01 (0.75-1.36)	0.954
Staying home	1.17 (0.90-1.51)	0.247	1.46 (1.06-2.02)	0.022	1.41 (1.17-1.70)	<0.001	**1.28 (0.96-1.70)**	**0.089***
Working from home	0.73 (0.57-0.94)	0.014	0.60 (0.42-0.83)	0.003	0.83 (0.70-1.01)	0.068	0.88 (0.66-1.17)	0.366
Avoiding travels outside one's own country or region	1.10 (0.86-1.41)	0.432	0.94 (0.69-1.28)	0.711	1.11 (0.91-1.34)	0.301	0.99 (0.75-1.31)	0.947
Outcome: Restless legs during sleep								
Change in work situation								
My work situation has not changed (reference)	-	-	-	-	-	-	-	-
I work/study from home	0.61 (0.14-8.05)	0.014	0.89 (0.49-1.59)	0.686	0.94 (0.69-1.28)	0.690	0.88 (0.50-1.57)	0.668
I was sent home without being able to work	1.13 (0.54-2.37)	0.739	0.85 (0.20-3.56)	0.822	1.67 (0.92-3.03)	0.090	0.85 (0.20-3.56)	0.822
I have taken a vacation or a leave of absence	1.07 (0.14-8.05)	0.944	3.25 (0.43-24.9)	0.255	Too few		Too few	
I was let go	Too few		1.03 (0.14-7.62)	0.977	2.78 (1.30-5.94)	0.008	1.18 (0.16-8.73)	0.874
I still go in to work, but my work situation has changed	1.23 (0.86-1.76)	0.263	1.40 (0.78-2.50)	0.259	1.07 (0.76-1.51)	0.682	1.16 (0.65-2.08)	0.611
Occupation								
Full time job (reference)	-	-	-	-	-	-	-	-
Part time job	0.87 (0.32-2.39)	0.788	1.09 (0.26-4.50)	0.906	1.01 (0.69-1.46)	0.969	1.29 (0.69-2.38)	0.423

Self employed	1.52 (0.90-2.59)	0.119	1.41 (0.61-3.29)	0.425	0.49 (0.18-1.32)	0.158	2.48 (1.05-5.87)	0.039
Student	1.11 (0.58-2.10)	0.755	0.71 (0.20-2.47)	0.589	1.42 (0.91-2.22)	0.123	0.84 (0.30-2.35)	0.733
Stay-at-home by choice	Too few		Too few		1.85 (0.66-5.22)	0.244	Too few	
Unemployed	0.74 (0.23-2.35)	0.605	1.26 (0.30-5.25)	0.751	0.96 (0.42-2.22)	0.931	2.34 (0.83-6.59)	0.109
Long-term sick leave/parental leave	2.66 (0.62-11.5)	0.191	Too few		1.95 (0.83-4.57)	0.125	Too few	
Retired	0.86 (0.42-1.77)	0.679	0.88 (0.30-2.60)	0.814	1.77 (1.13-2.76)	0.012	0.68 (0.23-2.02)	0.491
Other	1.81 (0.55-5.92)	0.326	1.47 (0.20-10.9)	0.707	1.21 (0.55-2.66)	0.630	2.28 (0.80-6.52)	0.123
Job type								
Academic (reference)	-	-	-	-	-	-	-	-
**Administration/Office work**	1.26 (0.84-1.91)	0.262	0.88 (0.43-1.82)	0.734	**0.96 (0.69-1.35)**	**0.829**	2.19 (1.20-3.99)	0.011
Sales/Service	1.09 (0.57-2.09)	0.801	1.76 (0.75-4.14)	0.195	1.46 (0.85-2.50)	0.166	2.98 (1.22-7.26)	0.016
Skilled worker	1.53 (1.02-2.31)	0.042	2.32 (1.30-4.16)	0.005	1.83 (1.11-3.01)	0.017	0.81 (0.19-3.57)	0.790
**Unskilled worker**	2.55 (1.39-4.68)	0.002	0.92 (0.21-3.94)	0.908	1.48 (0.77-2.84)	0.239	**3.75 (1.45-9.70)**	**0.006***
Other	1.78 (1.08-2.93)	0.023	1.17 (0.47-2.90)	0.742	1.56 (1.05-2.33)	0.029	2.29 (1.08-4.85)	0.031
**Stamina score (continuous)**	**0.95 (0.93-0.97)**	**<0.001***	**0.96 (0.92-0.99)**	**0.022***	**0.94 (0.93-0.96)**	**<0.001***	**0.93 (0.90-0.96)**	**<0.001***
Ever been prescribed anti-depressive medication	1.19 (0.70-2.00)	0.516	1.53 (0.73-3.20)	0.262	1.85 (1.37-2.50)	<0.001	1.42 (0.79-2.59)	0.241
Living with child(ren)								
No (reference)	-	-	-	-	-	-	-	-
Below school age (<5 years old)	0.34 (0.05-2.51)	0.291	2.18 (0.49-9.68)	0.304	0.40 (0.05-2.89)	0.362	Too few	
School age (5 – 18 years old)	1.12 (0.82-1.54)	0.469	1.24 (0.74-2.09)	0.411	0.79 (0.62-1.01)	0.062	0.82 (0.53-1.29)	0.395
Living with another adult (yes vs. no)	1.19 (0.85-1.66)	0.313	0.94 (0.57-1.55)	0.810	1.02 (0.79-1.32)	0.855	1.01 (0.63-1.63)	0.959
Educational level								
Elementary school (reference)	-	-	-	-	-	-	-	-
High school or vocational course	0.99 (0.59-1.65)	0.956	0.68 (0.34-1.31)	0.239	0.58 (0.41-0.82)	0.002	0.96 (0.46-2.00)	0.922
Short length education	0.96 (0.50-1.84)	0.903	0.39 (0.13-1.12)	0.081	0.63 (0.37-1.06)	0.082	1.34 (0.53-3.43)	0.536
Medium length education	0.75 (0.42-1.37)	0.353	0.53 (0.24-1.20)	0.127	0.43 (0.29-0.63)	<0.001	0.51 (0.22-1.19)	0.121
**Long length education**	0.50 (0.26-0.98)	0.042	0.23 (0.08-0.66)	0.006	**0.23 (0.13-0.42)**	**<0.001***	0.53 (0.19-1.50)	0.229
**Feeling worried about the COVID pandemic and cut-off from social experiences (yes vs. no)**	0.68 (0.42-1.09)	0.106	0.50 (0.21-1.14)	0.100	0.79 (0.53-1.19)	0.257	**1.92 (1.11-3.31)**	**0.019***
Daily alcohol intake during the pandemic (yes vs. no)	1.33 (0.69-2.59)	0.395	0.64 (0.15-2.65)	0.535	0.97 (0.47-2.03)	0.943	1.18 (0.36-3.86)	0.779
Smoking during the pandemic (yes vs. no)	1.19 (0.77-1.84)	0.426	1.15 (0.57-2.32)	0.692	1.56 (1.13-2.14)	0.007	0.75 (0.34-1.63)	0.467
**BMI during the pandemic (continuous)**	**1.04 (1.00-1.08)**	**0.038***	**1.11 (1.05-1.17)**	**<0.001***	**1.05 (1.03-1.08)**	**<0.001***	**1.06 (1.02-1.11)**	**0.002***
Specific COVID-precautions (yes vs. no)								
Frequently washing hands	1.02 (0.64-1.62)	0.931	0.78 (0.40-1.53)	0.477	1.11 (0.73-1.70)	0.622	1.57 (0.63-3.90)	0.329
Coughing or sneezing in the elbow	0.96 (0.69-1.34)	0.824	0.93 (0.55-1.56)	0.784	0.84 (0.63-1.23)	0.245	0.93 (0.53-1.64)	0.807
Using disposable handkerchiefs	0.67 (0.38-1.20)	0.181	1.38 (0.69-2.72)	0.361	1.29 (0.97-1.71)	0.079	1.25 (0.75-2.08)	0.398
Wearing disposable masks	Too few		1.01 (0.14-7.38)	0.991	1.66 (0.86-3.20)	0.131	0.57 (0.08-4.12)	0.576
Avoiding handshakes	0.89 (0.56-1.41)	0.608	0.89 (0.42-1.85)	0.746	0.62 (0.44-0.88)	0.008	1.14 (0.49-2.64)	0.755
Stopped greeting people with hugs and/or kisses on both cheeks	0.80 (0.57-1.12)	0.192	0.79 (0.46-1.35)	0.389	0.82 (0.60-1.11)	0.203	1.62 (0.78-3.40)	0.195
Limiting use of public transportation	0.92 (0.68-1.25)	0.612	0.93 (0.58-1.50)	0.767	0.84 (0.66-1.08)	0.172	1.25 (0.81-1.93)	0.317
Avoid places where many people gather	0.82 (0.62-1.08)	0.167	0.99 (0.64-1.54)	0.968	0.89 (0.70-1.14)	0.351	0.97 (0.61-1.54)	0.904
Staying home	0.88 (0.64-1.21)	0440	0.10 (0.75-1.93)	0.444	1.09 (0.85-1.39)	0.488	0.96 (0.61-1.52)	0.846

Working from home more often	0.67 (0.50-0.91)	0.009	0.70 (0.44-1.11)	0.128	0.66 (0.51-0.86)	0.002	0.96 (0.61-1.51)	0.860
Avoiding travels outside one's own country or region	0.86 (0.66-1.14)	0.293	0.93 (0.60-1.43)	0.731	1.03 (0.81-1.30)	0.826	0.96 (0.62-1.49)	0.864

Factors with a P-value<0.05 are marked in bold writing

Factors associated with health-related quality of life scores, stress levels, and quality of sleep measured during the COVID-19 pandemic

Multivariable linear regression analyses comprising all the variables that were displayed in Table 1 in the main manuscript were conducted to identify factors that affected MCS, PCS, stress score and quality of sleep during the COVID-19 pandemic (i.e. not the change in these outcomes). Moreover, multivariable logistic regression analyses comprising the same covariates were conducted to identify factors impacting quality of sleep during the pandemic. Factors affecting changes in scores (from before the pandemic to during) were displayed in the main manuscript.

Table S5. Linear regression analyses. Identification of characteristics associated with physical and mental health characteristics measured during the COVID-19 pandemic

	Men (N =12,150)		Women (N =14,303)	
	Coef. (95% CI)	P	Coef. (95% CI)	P
Outcome: Mental component score				
Age (continuous)	0.087 (0.075-0.099)	<0.001	0.097 (0.084-0.111)	<0.001
Change in work situation				
My work situation has not changed (reference)				
I work/study from home	-0.784 (-1.123- -0.444)	<0.001	-0.369 (-0.730- -0.008)	0.045
I was let go	-2.980 (-4.113- -1.848)	<0.001	-3.712 (-5.00- -2.421)	<0.001
I still go in to work, but my work situation has changed	-0.549 (-0.899- -0.199)	0.002		
Occupation				
Full time job (reference)				
Part time job				
Self employed	-0.609 (-1.138- -0.079)	0.024		
Stamina score	0.411 (0.385-0.437)	<0.001	0.421 (0.394-0.448)	<0.001
Ever been prescribed anti-depressive medication	-1.268 (-1.777- -0.757)	<0.001	-1.242 (-1.679- -0.806)	<0.001
Living with child(ren)				
No (reference)				
Below school age (<5 years old)				

School age (5 – 18 years old)	0.405 (0.089-0.722)	0.012		
Living with another adult (yes vs. no)			0.608 (0.294-0.923)	<0.001
Smoking during the pandemic (yes vs. no)	-0.743 (-1.175- -0.311)	0.001	-0.917 (-1.368- -0.466)	<0.001
BMI during the pandemic (continuous)	-0.050 (-0.080- -0.015)	0.006		
**Outcome: Physical component score**				
Age (continuous)	-0.054 (-0.063- -0.045)	<0.001	-0.048 (-0.058- -0.038)	<0.001
Change in work situation				
I still go in to work, but my work situation has changed	-0.500 (-0.766- -0.235)	<0.001		
Occupation				
Full time job (reference)				
Part time job			-0.726 (-1.001- -0.445)	<0.001
Job type				
Academic (reference)				
Sales/Service	-0.542 (-0.986- -0.099)	0.017	-0.745 (-1.244- -0.247)	0.003
Skilled worker	-1.053 (-1.389- -0.718)	<0.001	-1.058 (-1.673- -0.444)	0.001
Unskilled worker	-1.474 (-2.023- -0.924)	<0.001	-0.650 (-1.024- -0.276)	0.001
Other				
Stamina score	0.081 (0.061-0.100)	<0.001	0.045 (0.026-0.065)	<0.001
Ever been prescribed anti-depressive medication	-1.052 (-1.438- -0.665)	<0.001	-0.911 (-1.229- -0.592)	<0.001
Educational level				
Elementary school (reference)				
Long length education			0.562 (0.011-1.114)	0.046
Smoking during the pandemic (yes vs. no)	-0.376 (-0.703- -0.048)	0.025		
BMI during the pandemic (continuous)	-0.191 (-0.218- -0.164)	<0.001	-0.220 (-0.242- -0.199)	<0.001
**Outcome: Stress score**				
	Men (N=5,790)		Women (N=6.617)	
Age (continuous)	-0.055 (-0.065- -0.046)	<0.001	-0.072 (-0.082- -0.061)	<0.001
Change in work situation				
My work situation has not changed (reference)				
I was let go	2.761 (1.851-3.669)	<0.001	3.436 (2.443-4.428)	<0.001
I still go in to work, but my work situation has changed	0.554 (0.273-0.835)	<0.001	0.603 (0.325-0.883)	<0.001
Stamina score (continuous)	-0.491 (-0.512- -0.473)	<0.001	-0.508 (-0.528- -0.487)	<0.001
Occupation				
Full time job (reference)				
Part time job	0.714 (0.149-1.278)	0.013		
Self employed	0.455 (0.030-0.879)	0.036		
Ever been prescribed anti-depressive medication	1.351 (0.942-1.761)	<0.001	1.151 (0.816-1.486)	<0.001

Living with child(ren)				
No (reference)				
School age (5 – 18 years old)	0.428 (0.174-0.682)	0.001		
Living with another adult (yes vs. no)			-0.338 (-0.508- -0.096)	0.006
Smoking during the pandemic (yes vs. no)	0.518 (0.171-0.865)	0.003	0.519 (0.172-0.866)	0.003
BMI during the pandemic (continuous)	0.039 (0.011-0.068)	0.007	0.040 (0.018-0.063)	0.001

* The presented coefficients are those with a P-value<0.05 in the model comprising all covariates.

Table S6. Logistic regression analyses. Identification of characteristics associated with quality of sleep measured during the COVID-19 pandemic

	Men (N =12,150)		Women (N =14,303)	
	OR (95% CI)	P	OR (95% CI)	P
**Outcome: Insomnia (yes vs. no)**				
Age (continuous)	1.02 (1.01-1.02)	<0.001	1.01 (1.01-1.02)	<0.001
Change in work situation				
My work situation has not changed (reference)				
I was sent home without being able to work			1.31 (1.02-1.70)	0.038
Type of job				
Academic (reference)				
Skilled worker			1.33 (1.06-1.66)	0.014
Stamina score	0.94 (0.93-0.95)	<0.001	0.95 (0.94-0.96)	<0.001
Ever been prescribed anti-depressive medication	1.40 (1.16-1.69)	<0.001	1.37 (1.19-1.58)	<0.001
Living with child(ren)				
No (reference)				
School age (5 – 18 years old)			0.85 (0.76-0.85)	0.005
Living with another adult (yes vs. no)	0.86 (0.76-0.97)	0.014		
Educational level				
Elementary school (reference)				
High school or vocational course			0.80 (0.65-0.98)	0.028
Short length education			0.69 (0.53-0.90)	0.005
Medium length education			0.72 (0.58-0.90)	0.004
Long length education			0.71 (0.56-0.91)	0.008
BMI during the pandemic (continuous)	1.02 (1.01-1.03)	0.004	1.02 (1.01-1.03)	<0.001
**Outcome: Daytime fatigue (yes vs. no)**				
Age (continuous)	0.99 (0.98-0.99)	0.006	0.97 (0.96-0.99)	<0.001
Change in work situation				
My work situation has not changed (reference)				
I was let go	2.26 (1.21-4.22)	0.010	2.39 (1.42-4.04)	0.001
Occupation				
Full time job (reference)				
Part time job	1.86 (1.19-2.88)	0.006		
Stamina score	0.90 (0.89-0.92)	<0.001	0.91 (0.90-0.93)	<0.001
Ever been prescribed anti-depressive medication	1.92 (1.43-2.57)	<0.001	1.84 (1.50-2.25)	<0.001
Educational level				
Elementary school (reference)				
Long length education			0.58 (0.38-0.89)	0.011

Smoking during the pandemic (yes vs. no)	1.49 (1.12-1.98)	0.006	1.37 (1.09-1.72)	0.006
BMI during the pandemic (continuous)	1.07 (1.04-1.10)	<0.001	1.04 (1.02-1.05)	<0.001
**Outcome: Restless legs during sleep (yes vs. no)**				
	Men (N=5,790)		Women (N=6.617)	
Age (continuous)	1.04 (1.00-1.02)	0.005	1.03 (1.02-1.04)	<0.001
Change in work situation				
My work situation has not changed (reference)				
I was sent home without being able to work	1.59 (1.01-2.50)	0.043		
I was let go			2.12 (1.19-3.78)	0.011
Stamina score (continuous)	0.95 (0.94-0.97)	<0.001	0.95 (0.94-0.97)	<0.001
Occupation				
Full time job (reference)				
Part time job			0.73 (0.57-0.93)	0.011
Ever been prescribed anti-depressive medication	1.83 (1.36-2.47)	<0.001	1.44 (1.15-1.81)	0.002
Living with another adult (yes vs. no)	1.44 (1.12-1.86)	0.005	1.30 (1.07-1.58)	0.008
Educational level				
Elementary school (reference)				
Long length education			0.49 (0.31-0.76)	0.002
Smoking during the pandemic (yes vs. no)			1.38 (1.08-1.77)	0.011
BMI during the pandemic (continuous)	1.06 (1.04-1.09)	<0.001	1.06 (1.05-1.08)	<0.001

* The presented coefficients are those with a P-value<0.05 in the model comprising all covariates.
